# *Corynebacterium striatum* infections in oncologic patients: clinical spectrum, resistance profiles, and evidence of nosocomial transmission

**DOI:** 10.1128/jcm.00829-25

**Published:** 2025-09-19

**Authors:** Kenya Yukawa, Sohei Harada, Kohji Komori, Brian Hayama, Daisuke Ohkushi, Koichi Takeda, Taisuke Enokida, Akira Yarimizu, Kazumi Takehana, Kageto Yamada, Michihiko Goto, Kazuhiro Tateda

**Affiliations:** 1 Department of Microbiology and Infectious Diseases, Toho University Graduate School of Medicine 198311 , Tokyo, Japan; 2 Department of Infectious Diseases, Cancer Institute Hospital, Japanese Foundation for Cancer Research 13609, Tokyo, Japan; 3 Department of Microbiology and Infectious Diseases, Toho University School of Medicine, Tokyo, Japan; 4 Division of Collaborative Regional Infection Control, Department of Community Well-being, Toho University School of Medicine, Tokyo, Japan; 5 Clinical Laboratories, Cancer Institute Hospital, Japanese Foundation For Cancer Research 13609, Tokyo, Japan; 6 Division of Infectious Diseases, Department of Internal Medicine, University of Iowa Carver College of Medicine 12243 , Iowa City, Iowa, USA; Cleveland Clinic, Cleveland, Ohio, USA

**Keywords:** nosocomial transmission, whole-genome sequencing, *Corynebacterium striatum*

## Abstract

Among *Corynebacterium* species, *Corynebacterium striatum* is relatively frequently involved in invasive human infections. In this study, we collected clinical information from patients diagnosed with *C. striatum* infection at a single cancer center and performed antimicrobial susceptibility testing and whole-genome sequencing of the causative strains. Of the 51 patients with *C. striatum* infections, 15 (29.4%) had postoperative intra-abdominal infections, eight (15.7%) had postoperative skin and soft tissue infections of the neck, and eight (15.7%) had osteoarticular infections. In 15 patients, *C. striatum* was detected concomitantly with other bacteria. The median duration of antimicrobial therapy was 25 days, with 43 patients (84.3%) showing clinical improvement by day 14. The crude mortality up to 90 days post-diagnosis was 15.7%. Vancomycin was the most commonly used definitive therapy, and 40 patients (78.4%) received multiple antimicrobial agents. Oral minocycline was often administered in patients requiring long-term treatment. Antimicrobial susceptibility testing of 53 strains, including two strains from follow-up cultures from the same patient, showed that most strains were susceptible to daptomycin and tetracyclines. However, non-susceptibility was noted in two strains (3.8%) for daptomycin and four strains (7.5%) for tetracyclines, each associated with *psgA2* mutation and *tet*(W) carriage. Core-genome single-nucleotide polymorphism analysis of the strains and epidemiological reviews of the source patients identified three suspected clusters of nosocomial transmission involving seven patients. This study demonstrated that *C. striatum* can cause a range of infections in patients with underlying diseases, such as malignancy, and that nosocomial spread of this pathogen may also occur.

**IMPORTANCE:**

The use of matrix-assisted laser desorption/ionization time-of-flight mass spectrometry, employed for bacterial species identification in this study, has enhanced the recognition of *Corynebacterium striatum* as an important human pathogen in clinical microbiology laboratories. Our study demonstrated that *C. striatum* is associated with various healthcare-associated infections, including those requiring prolonged antimicrobial therapy, and that nosocomial transmission of this pathogen can result in the development of infections. In addition, several agents other than vancomycin, such as teicoplanin, tetracyclines, and trimethoprim/sulfamethoxazole, have demonstrated favorable activities. The results of this study indicate the need for further research on the mechanisms and modes of nosocomial transmission of *C. striatum*, as well as the clinical efficacy of alternative agents to vancomycin, particularly those suitable for prolonged treatment, given the potential side effects associated with vancomycin use.

## INTRODUCTION

*Corynebacterium striatum* is a gram-positive bacillus belonging to the genus *Corynebacterium* and recognized as a commensal bacterium of human mucosa and skin (1). Non-diphtheriae *Corynebacterium* species are generally regarded as low pathogenic to humans and are often considered contaminants when detected in clinical culture samples. However, *C. striatum* is frequently involved in invasive infections among these species. In a study evaluating the clinical significance of *Corynebacterium* spp. detected in blood cultures, the frequency of true bacteremia caused by *C. striatum* was found to be 70.1%, which was significantly higher than that by other *Corynebacterium* species (2). In recent years, with the increasing use of matrix-assisted laser desorption/ionization time-of-flight mass spectrometry (MALDI-TOF MS) in microbiology laboratories, the frequency and accuracy of identification of *Corynebacterium* spp. from clinical specimens have increased, and the clinical importance of infections caused by these species has been emphasized (3).

Invasive infections caused by *C. striatum* are reported to occur primarily in patients with underlying diseases such as malignancy, chronic obstructive pulmonary disease, and diabetes (2, 4, 5). Although respiratory and catheter-associated bloodstream infections have been frequently reported infections caused by *C. striatum*, there is insufficient information on the spectrum of infections. *C. striatum* is not routinely monitored as a healthcare-associated pathogen, and the epidemiology of infections caused by this organism in hospitalized patients is largely unknown, although previous reports have suggested its potential nosocomial spread. However, the frequency and modes of transmission are not well understood.

*C. striatum* is generally a multidrug-resistant organism, and vancomycin is most commonly used for the treatment of related infections. Reflecting its multidrug resistance, *C. striatum* harbors various antimicrobial resistance genes (6). Nevertheless, the influence of each antimicrobial resistance gene on antimicrobial susceptibility has not yet been fully evaluated. Given that vancomycin is a parenteral agent associated with toxicity concerns, such as acute kidney injury, the development of alternative therapeutic options is warranted. However, the clinical efficacy of other agents has not yet been assessed.

To address these knowledge gaps, this study aimed to clinically and microbiologically characterize cases of infection caused by *C. striatum* at a large cancer center in Japan. Information on patient characteristics and the therapeutic course was obtained from medical records, and antimicrobial susceptibility testing and whole-genome sequencing of the isolated strains were conducted to analyze the relationship between the presence of resistance genes and antimicrobial susceptibility. In addition, the possibility of nosocomial transmission was investigated by integrating genomic similarity analyses of the strains with epidemiological reviews of the source patients.

## MATERIALS AND METHODS

### Study design, setting, and eligibility criteria

This study was performed at the Cancer Institute Hospital, Japanese Foundation for Cancer Research. The hospital is a single building with 20 operating rooms clustered in one location, one 10-bed intensive care unit (ICU), separate 16 wards (Ward A through Ward P), and a total of 686 beds. The detection of *Corynebacterium* spp. in blood and other specimens from sites not typically colonized by commensals was always reported to clinicians. However, detection in other specimens was reported only when the bacterial load reached ≥10^4^ colony-forming units/mL in urine, or when a clear predominance was observed based on the Gram staining or culture growth.

Patients with *Corynebacterium* infections who received care involving the Department of Infectious Diseases through consultation at the hospital, between January 2015 and July 2024, were selected as candidates. The strains detected in these patients were frozen-stored, and the bacterial species were identified using MALDI Biotyper (Bruker Daltonics, Bremen, Germany) at Toho University. This study included cases caused by strains identified as *C. striatum* with a score value of ≥2.000. If a patient had experienced multiple episodes of *C. striatum* infection during the study period, only the first episode was included.

### Clinical data collection

The following clinical information was systematically collected from electronic medical records: demographics, setting in which the infection occurred as defined in a previous study (classified into three categories: community-acquired, healthcare-associated, or hospital-acquired) (7), Charlson comorbidity index (8), HIV infection, immunocompromising conditions (Table S1), history of antimicrobial use and surgery within 30 days before the diagnosis, presence of intravascular devices or other artificial devices, Pitt bacteremia score (9), and site of infection. The diagnosis of *C. striatum* infection was determined by an infectious disease physician based on the following criteria: (i) growth of *C. striatum* in multiple sets of blood cultures accompanied by clinical findings, imaging findings, or a positive local culture specimen suggestive of the source of bacteremia; (ii) clinical or imaging findings suggestive of the localized infection, with detection of *C. striatum* from specimens not typically colonized by commensals or as a monomicrobial culture result; or (iii) clinical or imaging findings suggestive of localized infection with *C. striatum* identified in culture, and the infectious disease specialist deemed antimicrobial therapy necessary. Information on empiric and definitive antimicrobial therapy until day 90 was also collected. Empiric therapy was defined as the administration of antimicrobial agents with activity against gram-positive bacteria between the collection of culture specimens in which *Corynebacterium* was detected and subsequent identification of the organism. Definitive therapy was defined as the administration of antimicrobial agents to treat *Corynebacterium* infections based on the susceptibility report of the isolate after organism identification. Clinical improvement by day 14, defined as a composite of fever resolution, improvement in local inflammatory findings, and documented negative culture results for cases in which follow-up culture tests were performed, was evaluated as an outcome measure. Laboratory-confirmed *Clostridioides difficile* infection and all-cause mortality by day 90 were also assessed. Day 0 was defined as the day on which the first culture specimen with *Corynebacterium* growth was collected.

Additionally, the hospital admission histories of patients within 30 days before the detection of *C. striatum* were investigated. In the patient groups with high genomic similarity, as detected by the single-nucleotide polymorphism (SNP) analysis, hospital admission histories from 6 months before the month when the first strain in each group was detected, up to the month when the last strain was detected, were also reviewed.

### Analysis of *C. stratum* strains

Antimicrobial susceptibility testing and whole-genome sequencing were conducted for all *C. striatum* strains. For two eligible patients, two strains from the same patient were stored because of recurrence of the infection after a long interval or the development of daptomycin resistance. The strains collected later (TUM25355 and TUM25358) were also included in the strain analysis.

We used the broth microdilution method for antimicrobial susceptibility testing, as outlined in the CLSI M45-ED3 guidelines (10). Cation-adjusted Mueller-Hinton broth supplemented with lysed horse blood was used, and the calcium concentration was adjusted to 50 µg/mL for daptomycin susceptibility testing. Minimum inhibitory concentrations (MICs) were determined for penicillin (Sigma-Aldrich Co. LLC, St. Louis, MO, USA) (range: 0.06–64 µg/mL), ampicillin (FUJIFILM Wako Pure Chemical Corporation, Osaka, Japan) (0.06–64 µg/mL), ampicillin/sulbactam (FUJIFILM Wako Pure Chemical Corporation) (0.06/0.03–64/32 µg/mL), ceftriaxone (Sigma-Aldrich Co. LLC) (0.12–16 µg/mL), cefepime (United States Pharmacopeia, Rockville, MD, USA) (0.12–16 µg/mL), meropenem (FUJIFILM Wako Pure Chemical Corporation) (0.06–8 µg/mL), levofloxacin (LKT Laboratories, Inc., St. Paul, MN, USA) (0.03–64 µg/mL), ciprofloxacin (LKT Laboratories, Inc.) (0.03-64 µg/mL), tetracycline (Sigma-Aldrich Co. LLC) (0.03–64 µg/mL), doxycycline (Tokyo Chemical Industry Co., Ltd., Tokyo, Japan) (0.03–64 µg/mL), minocycline (Tokyo Chemical Industry Co., Ltd.) (0.03–64 µg/mL), erythromycin (FUJIFILM Wako Pure Chemical Corporation) (0.12–16 µg/mL), clindamycin (Tokyo Chemical Industry Co., Ltd.) (0.12–16 µg/mL), gentamicin (FUJIFILM Wako Pure Chemical Corporation) (0.25–32 µg/mL), trimethoprim (FUJIFILM Wako Pure Chemical Corporation)/sulfamethoxazole (Shionogi & Co., Ltd., Osaka, Japan) (2.3/0.12–152/8 µg/mL), vancomycin (Sigma-Aldrich Co. LLC) (0.12–16 µg/mL), teicoplanin (Aventis Pharma, Tokyo, Japan) (0.12–16 µg/mL), daptomycin (Tokyo Chemical Industry Co., Ltd.) (0.03-4 µg/mL), and linezolid (Tokyo Chemical Industry Co., Ltd.) (0.06-8 µg/mL). *Streptococcus pneumoniae* ATCC 49619, *Escherichia coli* ATCC 25922 for gentamicin and minocycline, and *E. coli* ATCC 35218 for ampicillin-sulbactam were used as quality control strains.

Whole-genome sequencing of *C. striatum* strains was performed using MiSeq (Illumina, San Diego, CA, USA). DNA was extracted from the bacterial cells using the bead-beating method with EZ-Beads (Promega K.K., Tokyo, Japan), followed by processing with a combination of magLEAD 6gC and MagDEA Dx SV, using the PS protocol (Precision System Science Co., Ltd., Chiba, Japan). DNA libraries were prepared using Illumina DNA Prep (Illumina) and sequenced with MiSeq with 300 bp paired-end reads. Raw reads generated by MiSeq were trimmed to remove adapter sequences, as well as those not meeting a quality score threshold of Q30 or higher using the Trimmomatic tool (version 0.39) with the following parameters: LEADING:30, TRAILING:30, SLIDINGWINDOW:4:15, MINLEN:100, and HEADCROP:5 (11). Trimmed reads were assembled using SPAdes (version 3.15.4) with paired-end reads (12).

The average nucleotide identity (ANI) of the obtained genomes and the genome of the reference strain *C. striatum* NBRC 15291^T^ (GenBank accession number: NZ_BJLD00000000) were calculated using FastANI (version 1.33), and genetic identification of the bacterial species was performed using an ANI threshold of 95% or higher (13).

Antimicrobial resistance genes of *C. striatum* strains were identified using ResFinder (version 4.6.0) (http://genepi.food.dtu.dk/resfinder). In addition, the class A β-lactamase gene (*bla*_Coryne-A_) and the class C β-lactamase gene (*ampC*), both of which have been reported across multiple *Corynebacterium* species in previous studies, were identified manually using the Basic Local Alignment Search Tool (https://blast.ncbi.nlm.nih.gov/Blast.cgi) (14). Mutations in the quinolone resistance-determining regions (QRDRs) of *gyrA* were identified using the nucleotide sequence of *gyrA* from *C. striatum* ATCC 6940 strain (GenBank accession number: AY559038) as the reference (14). Additionally, the daptomycin resistance mechanism was analyzed by identifying mutations in the phosphatidylglycerol synthase gene (*pgsA2*), using the nucleotide sequence of *pgsA2* in *C. striatum* WP1a strain (GenBank accession number: NZ_SBIF00000000) as the reference (15).

Genomic similarity of *C. striatum* strains was assessed using core-genome SNP analysis. The genomic sequence of *C. striatum* FDAARGOS_1115 strain (GenBank accession number: NZ_CP068158) was used as the reference. Genome data of *C. striatum* strains obtained in this study were aligned to the genome sequences of the reference strain using the Burrows-Wheeler Aligner with the “SW” algorithm (16). Core-genome alignments were extracted using SAMtools (version 1.21) mpileup, with base alignment quality adjustment disabled and the reference genome explicitly specified (17). SNPs and consensus sequences were called using VarScan (version 2.3.9) with the mpileup2cns function and default parameters (18). A temporal phylogenetic tree was constructed using the maximum likelihood method with PhyML (version 3.3.20241207) and general time reversible (GTR) substitution model, with 100 bootstrap replicates (19). This tree was used as the initial input for ClonalFrameML (version 1.12) to infer homologous recombination events that introduced DNA fragments from strains outside the phylogenetic clade, thereby generating a recombination-corrected clonal phylogeny (20). A phylogenetic tree based on core-genome SNPs, excluding homologous recombination regions inferred by ClonalFrameML, was reconstructed using the maximum likelihood method implemented in RAxML (version 8.2.12) with the GTR substitution model and 1,000 bootstrap replicates (21). All phylogenetic trees were visualized using FigTree (version 1.4.4). The number of SNPs in the core genome was calculated using Snp-dists (version 0.7.0) (https://github.com/tseemann/snp-dists). For the isolates belonging to the 11 groups with high genomic similarity identified in the first analysis, core-genome SNP analysis was performed again, restricting the analysis to isolates within each clade to expand the range of nucleotide sequences included in the core genome. In this study, the threshold for possible nosocomial transmission between strains was set at 20 or fewer SNPs, or less than two SNPs per month between the detection dates of the strains.

## RESULTS

### Study population

Of the 61 cases diagnosed with *Corynebacterium* infections, 51 cases of *C. striatum* infection identified using the MALDI Biotyper were included. Twelve cases were diagnosed based on multiple positive blood cultures, 16 involved specimens from sites not typically colonized by commensals (pleural fluid, ascites, abscess contents, surgical specimens, etc.), and 23 were clinically diagnosed based on the isolation of *C. striatum* from other specimens (sputum, urine, open wounds, etc.) (Table S2). In eight of these patients, *C. striatum* was detected with other bacteria. Of the 23 specimens that could be contaminated with commensals, *C. striatum* was detected as the only pathogen in 16 specimens. In the remaining seven specimens, *C. striatum* was detected as part of polymicrobial culture results.

As the study site was a cancer center, all patients had a diagnosis of malignancy. The cancer types were diverse, with head and neck cancer being the most common (12 cases, 23.5%), and 27 cases (52.9%) had metastatic tumors (Table S3). Hospital-acquired infections accounted for 39 cases (76.5%), healthcare-associated infections for 11 cases (21.6%), and community-acquired infections for only one case (2.0%). Thirty patients (58.8%) had a history of surgery within 30 days before the collection of the culture specimen in which *C. striatum* was detected. Forty-three patients (84.3%) had non-vascular prosthetic devices, with urinary tract prostheses, percutaneous drains, and osteoarticular prostheses being the most common. Antimicrobials were used within 30 days before *C. striatum* detection in 47 patients (92.2%), including 33 patients (64.7%) who received antimicrobials lacking activity against *C. striatum*.

**Fig 1:**
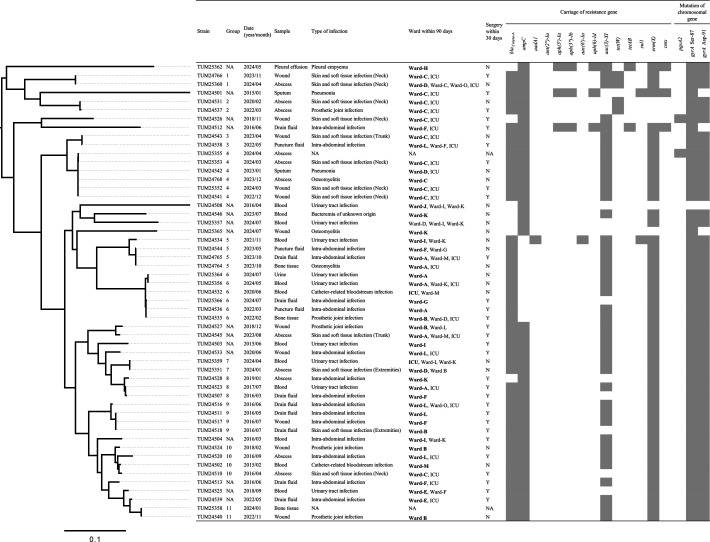
Phylogenetic relationships based on core-genome SNP analysis combined with clinical data and resistance gene profiles of the strains. The core-genome size was 11.8% (342,256 bp/2,904,831 bp) of the genomic sequences of the reference strain, *Corynebacterium striatum* FDAARGOS_1115 (GenBank accession number: NZ_CP068158). The presence of resistance genes in each strain is indicated with a gray box. The hospital ward of the patient on the day of culture specimen collection is shown in bold. TUM25357 was detected in a specimen collected in an outpatient setting. Abbreviation: Y, Yes; N, No; NA, not applicable.

Culture specimens positive for *C. striatum* were collected from patients admitted to diverse hospital wards; however, Ward C (*n* = 11), Ward A (*n* = 7), and Wards B, F, and L (*n* = 5) were predominant (Table S3). Within 30 days of diagnosis, many patients were admitted to multiple wards, including a short postoperative ICU stay (Fig. 1).

### Characteristics, treatment, and prognosis of *C. striatum* infection

Sixteen patients (31.4%) had intra-abdominal infections, and all but one had a history of surgery within 30 days preceding the infection, which developed as organ/space surgical site infections (SSIs), according to the National Healthcare Safety Network SSI criteria (22) (Table S3). There were 12 patients (23.5%) with skin and soft tissue infections, of which nine had a history of surgery within 30 days preceding the infection, with eight involving neck lesions after head and neck surgery. There were cases of prosthetic joint infections (*n* = 5), osteomyelitis (*n* = 3), and pleural empyema (*n* = 1), which required prolonged antimicrobial treatment. Three cases of prosthetic joint infections were associated with surgery within 30 days preceding the infection.

The median duration of antimicrobial therapy was 25 days (interquartile range: 15–44.5 days). Twenty-three patients received treatment for 30 days or more, and four patients did not complete antimicrobial therapy by day 90. While 41 patients received empirical therapy (predominantly glycopeptides or daptomycin), treatment was initiated only after the detection of *C. striatum* in 10 patients. Vancomycin was the most commonly used definitive therapy, followed by oral minocycline. Forty patients received multiple antimicrobial agents during their therapeutic courses, and a switch from intravenous to oral agents was often implemented in cases requiring long-term treatment.

The median Pitt bacteremia score was 0. Forty-three patients (84.3%) showed clinical improvement within 14 days, and only eight patients (15.7%) died within 90 days. There were no cases of *C. difficile* infection.

### Genetic identification of bacterial species

All 53 strains were genetically identified as *C. striatum*, with an ANI of >95% compared to the genome of the type strain (Table S2), confirming the accuracy of identification by the MALDI Biotyper.

### Antimicrobial susceptibility and resistance gene profiles of *C. striatum* strains

All strains were susceptible to vancomycin and linezolid (Table 1; Table S2). Susceptibility to teicoplanin could not be assessed because of the lack of breakpoints; however, all strains had MICs that were the same or slightly lower than those of vancomycin.

**TABLE 1:** Antimicrobial susceptibility of 53 *Corynebacterium striatum* strains^*b*^

	Susceptibility^*a*^			MIC_50_ (μg/mL)	MIC_90_ (μg/mL)
	Susceptible	Intermediate	Resistant		
Penicillin	1 (1.9)	6 (11.3)	46 (86.8)	>64	>64
Ampicillin	NA	NA	NA	>64	>64
Ampicillin/sulbactam	NA	NA	NA	>64/32	>64/32
Ceftriaxone	0 (0)	1 (1.9)	52 (98.1)	>16	>16
Cefepime	6 (11.3)	1 (1.9)	46 (86.8)	>16	>16
Meropenem	7 (13.2)	0 (0)	46 (86.8)	>8	>8
Tetracycline	49 (92.5)	0 (0)	4 (7.5)	0.25	2
Minocycline	NA	NA	NA	£0.03	0.5
Doxycycline	49 (92.5)	0 (0)	4 (7.5)	0.12	2
Vancomycin	53 (100)	NA	NA	0.5	0.5
Teicoplanin	NA	NA	NA	£0.25	0.5
Daptomycin	51 (96.2)	NA	NA	0.06	0.5
Linezolid	53 (100)	NA	NA	0.25	0.5
Gentamicin	49 (92.5)	1 (1.9)	3 (5.7)	2	4
Ciprofloxacin	1 (1.9)	0 (0)	52 (98.1)	>64	>64
Levofloxacin	NA	NA	NA	>64	>64
Erythromycin	5 (9.4)	0 (0)	48 (90.6)	>16	>16
Clindamycin	3 (5.7)	5 (9.4)	45 (84.9)	>16	>16
Trimethoprim/sulfamethoxazole	42 (79.2)	NA	11 (20.8)	0.5/9.5	>8/152

^*a*^ The results are interpreted according to the CLSI guidelines (10). Data are presented as number (%).

^*b*^ MIC, minimum inhibitory concentration; NA, not applicable.

Two strains were nonsusceptible to daptomycin, both with mutations leading to premature termination of protein synthesis in *pgsA2*, which encodes phosphatidyl glycerol synthase. One of the strains (TUM25355) was detected 12 days after the detection of the daptomycin-susceptible strain with the wild-type *pgsA2* gene (TUM25353) following 10 days of daptomycin administration in a patient admitted to Ward C (Fig. 1). Although another daptomycin-nonsusceptible strain (TUM24526) was detected in a patient from Ward C, the overall genomic background of this strain differed from that of TUM25355. However, the patient received daptomycin for 6 days within 30 days preceding the detection of the isolate. Besides these patients, only one patient (TUM24503) had a history of daptomycin administration within 30 days (for 1 day).

The 46 strains harboring *bla*_Coryne-A_ were resistant to all β-lactams. All seven strains lacking *bla*_Coryne-A_ were susceptible to meropenem; however, some were nonsusceptible to other β-lactams. There were no differences between MICs of ampicillin and ampicillin/sulbactam, despite most strains carrying the class A β-lactamase gene. The truncation of *ampC* identified in ten strains did not appear to be associated with β-lactam susceptibility.

Although the four strains harboring *tet*(W) were resistant to tetracycline and doxycycline, the remaining 49 strains were susceptible to both agents. MICs of the two strains harboring *tetAB* were slightly higher for tetracycline within the susceptible range; however, the MICs for doxycycline and minocycline were lower in these strains. Susceptibility to minocycline could not be assessed owing to the lack of breakpoints, but its MICs tended to be the lowest among the three agents.

Forty-two strains were found to be susceptible to trimethoprim/sulfamethoxazole. Of the 11 trimethoprim/sulfamethoxazole-resistant isolates, only one harbored *sul1*, and the other strain harboring *sul1* was susceptible to trimethoprim/sulfamethoxazole. All strains except one were resistant to ciprofloxacin, and these resistant strains had Ser-87 and/or Asp-91 mutations in *gyrA*. Four isolates were nonsusceptible to gentamicin; however, the association between susceptibility and the presence of aminoglycoside-modifying enzyme genes was unclear.

### Assessment for nosocomial transmission of *C. striatum* strains

Although the strains collected in this study were genetically diverse, as revealed by core-genome SNP analysis, 11 groups consisting of genomically similar strains were identified (Fig. 1). Several resistance genes were associated with specific genetic lineages. For example, *tet*(W) was present in only four strains from Groups 1 and 2 with a common origin, and truncation of *ampC* was detected in only 10 strains from Groups 5 and 6 with another common origin. In contrast, two daptomycin-resistant strains were unrelated.

**Fig 2:**
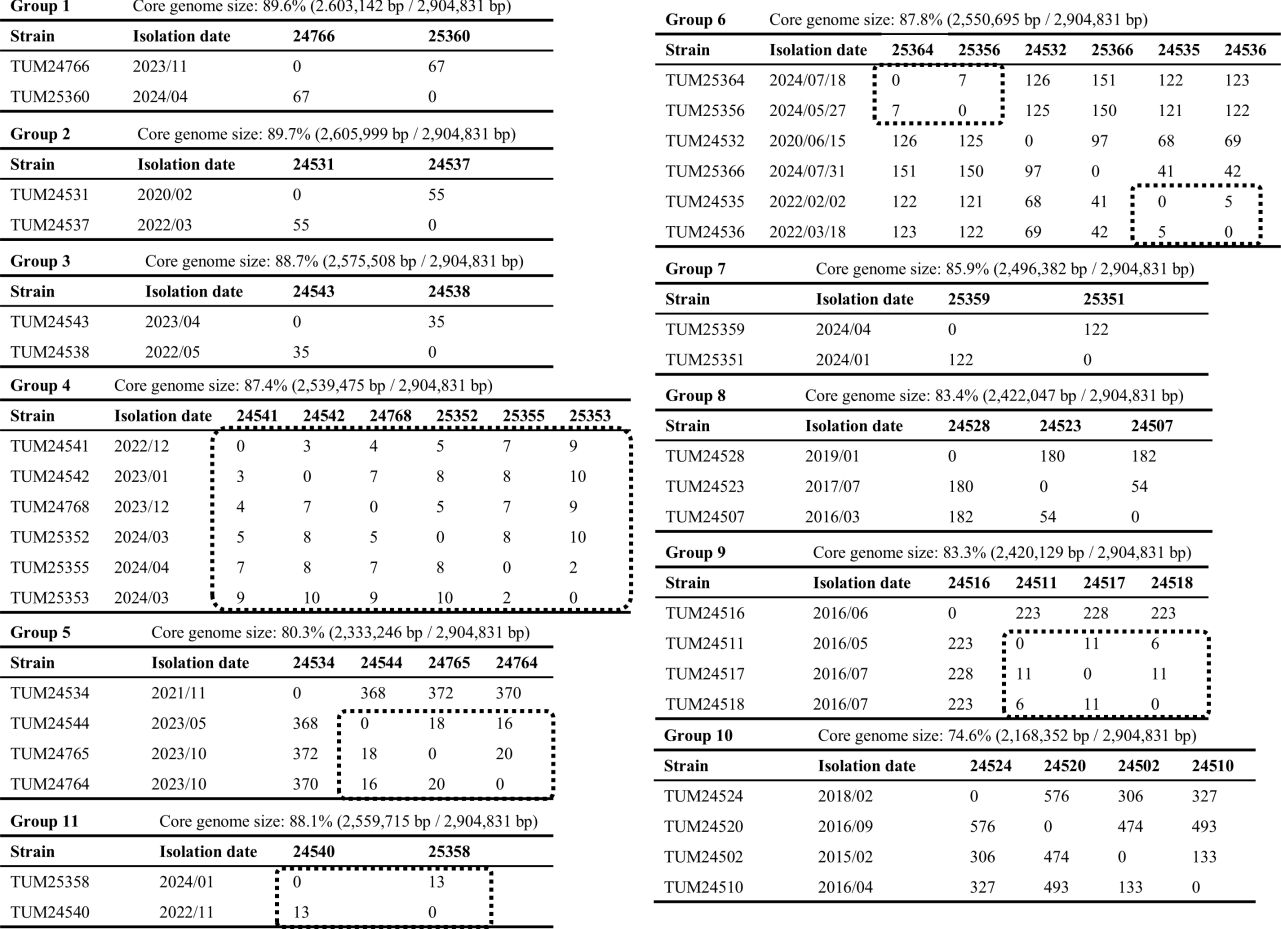
SNP differences between strains in each group. The core genome size of each group of strains was determined using *Corynebacterium striatum* FDAARGOS_1115 (GenBank accession number: NZ_CP068158) as the reference. Clusters of strains with an SNP difference of 20 or less are highlighted with dotted lines.

**Fig 3:**
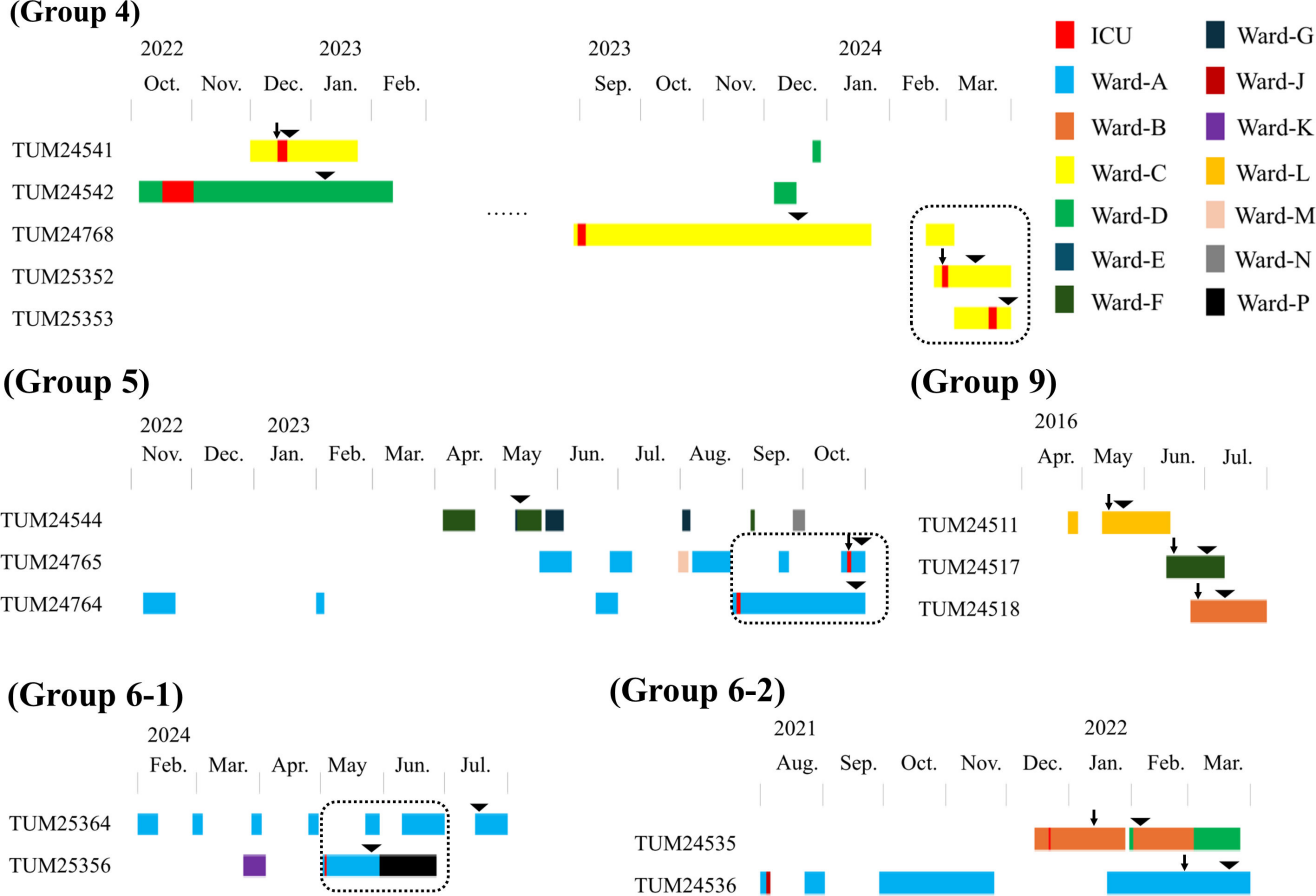
Hospitalization history of patients from whom strains with high genomic similarity were isolated. The duration of stay in each ward is indicated by colored bars. The black triangle indicates the date on which the strain was isolated. Black arrows indicate the date of surgery within 30 days of strain detection. Dotted lines indicate the periods when patients for whom strains with high genomic similarity were detected were admitted simultaneously to the same ward.

Core-genome SNP analysis limited to strains belonging to the same group identified six clusters of strains suspected of nosocomial transmission (Fig. 2). As one of the six clusters that consisted of two strains detected in the same patient (TUM25358 and TUM24540) with recurrent prosthetic joint infection, we reviewed the hospitalization histories of the source patients of the strains belonging to the remaining five clusters and identified seven patients in three clusters who shared the same ward, but in different rooms, at the same timeframe (TUM24768, TUM25352, and TUM25353; TUM24765 and TUM24764; TUM25364 and TUM25356) (Fig. 3).

## DISCUSSION

In this study, we examined the clinical and microbiological characteristics of 51 patients with *C. striatum* infection diagnosed at a single cancer center during a 10-year study period. *C. striatum* caused a wide variety of infections, including surgical site infections of the neck and abdominal cavity and osteoarticular infections, for which prolonged antimicrobial therapy is often necessary. Most strains were multidrug-resistant but showed favorable susceptibility to vancomycin, daptomycin, linezolid, tetracyclines, and trimethoprim-sulfamethoxazole. Resistance to several antimicrobial agents was distinctly associated with the presence of relevant resistance genes. Several probable cases of nosocomial transmission were documented.

In this study, most *C. striatum* infections were healthcare-associated or nosocomial infections. Since this study was conducted at a cancer center, patients had various underlying malignancies, often with a history of recent surgery or prosthesis implantation. The finding that *C. striatum* infections occurred primarily in hospitalized patients and those with prosthetic devices was consistent with that of previous studies (23). A unique feature of this study was that many patients presented with surgical site infections in the neck or abdominal cavity. In previous studies, respiratory infections, catheter-related bloodstream infections, and infective endocarditis were common infections caused by *C. striatum* (2, 4, 5, 24, 25). Additionally, osteoarticular infections, including those associated with prostheses, were observed in eight cases. Although osteoarticular infections caused by *C. striatum* have previously been reported, the frequency of these infections has been unclear (23, 26, 27). The results of this study may suggest a higher frequency of difficult-to-treat infections by *C. striatum* in patients with malignancies. The identification of *C. striatum* detected as the causative agent of *Corynebacterium* infection in 51 of the 61 cases reaffirmed the clinical importance of this species within the genus. Although all patients had cancer and more than half of them had metastatic tumors, only eight patients (15.7%) had died of any cause by day 90. This was lower than the reported 90-day mortality rate in a study that included only *C. striatum* bacteremia (34%) (2).

This study also provides insights into the treatment practices for *C. striatum* infections at our institution. The duration of antimicrobial therapy in patients with *C. striatum* infections was prolonged in many cases. In intra-abdominal infections, which accounted for 16 cases (31.4%), antimicrobial therapy is generally recommended for only 4–8 days once adequate source control is achieved (28). However, 15 of 16 intra-abdominal infections in this study were post-surgical, for which percutaneous or surgical drainage could be challenging, and source control was often incomplete. In such cases, the duration of antimicrobial therapy is individualized and often prolonged. SSIs after head and neck cancer surgery, which accounted for eight cases (15.7%), were postoperative complications, which could lead to serious consequences, such as reconstructive tissue loss and deep neck infection. The duration of antimicrobial therapy is not well established in these cases, and prolonged therapy is often employed (29). In addition, osteomyelitis and prosthetic joint infection, which accounted for eight cases (15.7%), are recommended to be treated for 6 weeks or longer (30, 31). In patients treated with prolonged parenteral antimicrobial therapy, a subsequent oral step-down therapy was often prescribed, with oral minocycline being the most commonly used. Minocycline and doxycycline have also been used as long-term suppressive therapies in a previous case series of prosthetic joint infections, despite insufficient validation of their clinical efficacy (26). Linezolid is another option; however, frequent side effects and high cost may preclude its use.

Most *C. striatum* strains in this study were multidrug-resistant, as is generally recognized (14, 32). Nevertheless, vancomycin and linezolid were active against all strains and appeared to be reliable treatment options. Although the CLSI and EUCAST did not establish a susceptibility breakpoint for teicoplanin, it showed the same or slightly lower MIC than that of vancomycin. Daptomycin showed a high overall susceptibility rate; however, two nonsusceptible strains (3.8%) were observed. These strains harbored *pgsA2* mutation, which is a well-recognized mechanism of daptomycin resistance in *C. striatum* (15). One of these strains acquired this mutation after a short duration of daptomycin treatment. Although the other daptomycin-nonsusceptible strain had a distinct genomic background and was the first strain detected in the patient, the patient also had a history of prior daptomycin administration. *C. striatum* has a higher rate of resistance development with *in vitro* daptomycin exposure than other non-diphtheriae *Corynebacterium*, and 4 of 11 cases in a previous case series of *C. striatum* bacteremia treated with daptomycin developed resistance after short-term (minimum 3 days) treatment, as in the present study (33, 34).

Tetracyclines, trimethoprim/sulfamethoxazole, and fluoroquinolones are oral antimicrobial alternatives against *Corynebacterium* infections if the isolates are susceptible. All strains except four (7.5%) with *tet*(W) were susceptible to tetracyclines. This suggests that these agents are promising options for oral step-down therapy as they have been selected in many cases. However, further validation of their clinical efficacy and relevance of the current breakpoints is warranted. The development of breakpoints for minocycline is also required. Resistance rates were 58.3% for tetracycline and 28.1% for doxycycline in strains collected from a single center in China (*n* = 192) and 17.5% for doxycycline in strains collected from a single center in Tunisia (*n* = 63), suggesting regional differences in susceptibility (14, 32). Trimethoprim/sulfamethoxazole, with only few reports on susceptibility data, may also be a candidate for oral step-down therapy, owing to its favorable susceptibility. Fluoroquinolone resistance observed in most strains in this study was consistent with a report from China, whereas strains detected in Tunisia showed comparatively favorable susceptibility (14, 32).

Core-genome SNP analysis identified six clusters of strains, each consisting of two to six strains, with an SNP difference of 20 or less. One group consisted of two strains (TUM24540 and TUM25358) derived from a prosthetic joint infection and its relapse in the same patient, with detection dates 14 months apart and an SNP difference of 13. It is challenging to establish a threshold for SNPs to be considered the same clone in long-term observations, as in this study, because of insufficient information on the rate of mutation accumulation in the *C. striatum* genome. We set a conservative criterion of 20 or less, and less than 2 per difference in detection months based on the criteria applied to other bacteria and the results from the same patients described above (35, 36). However, no strain pairs with SNP differences > 20 fulfilled less than two per difference in detection months. In three of the five clusters of strains derived from different patients, there was a history of hospitalization in the same ward during the same period before the strain was detected in the source patients, suggesting nosocomial transmission. However, no history of hospitalization in the same room was documented in any patient pair. Outbreaks of *C. striatum* in ICUs and respiratory wards have been repeatedly reported, and a recent report from China demonstrated the spread of multiple clones of *C. striatum* isolates across multiple hospital wards (25, 32, 37). Although the epidemiology of *C. striatum* in nosocomial settings is largely unknown, it may cause a nonnegligible healthcare burden given its multidrug-resistant nature.

This study had several limitations. First, this was a single-center study that included only cases in which infectious disease physicians were involved in patient care. In addition, the characteristics of the infections reflected those of the institution, a cancer center that admits many perioperative patients. However, it is still generalizable that *C. striatum* can cause various infections that often require prolonged treatment and provides novel insights into the potential risk of nosocomial transmission. Second, in some cases, it was difficult to determine whether the *C. striatum* strains were true pathogens. However, the patients in this study were deemed to require treatment by infectious disease specialists, making our findings meaningful for antimicrobial stewardship. Third, the specific mode of nosocomial transmission of *C. striatum* strains remains unknown. Transmission among inpatients in the same ward but in different rooms suggests the possibility of transmission via healthcare professionals’ hands, shared medical instruments, and hospital environments; however, surveillance cultures of medical instruments and hospital environments were not performed.

In conclusion, we described the clinical and microbiological characteristics of *C. striatum* infections at a large cancer center and identified the possible nosocomial transmission of multidrug-resistant *C. striatum*. Further studies are warranted to gain more insights into the characteristics and therapeutic options against *C. striatum*, which has been increasingly recognized as an important pathogen in recent years.

## Data Availability

Genome sequences have been deposited in the NCBI database under BioProject accession numbers PRJNA1237426.

## SUPPLEMENTAL MATERIAL

Table S1 jcm.00829-25-s0001.xlsx Definitions of immunocompromising conditions in this study.

Table S2 jcm.00829-25-s0002.xlsx Profiles of *Corynebacterium striatum* strains in this study.

Table S3 jcm.00829-25-s0003.xlsx Characteristics of patients with *Corynebacterium striatum* infection.

ASM does not own the copyrights to Supplemental Material that may be linked to, or accessed through, an article. The authors have granted ASM a non-exclusive, world-wide license to publish the Supplemental Material files. Please contact the corresponding author directly for reuse.
